# In situ analysis of Her2 DNA and RNA in retinoblastoma and adjacent retina

**DOI:** 10.18632/oncoscience.489

**Published:** 2019-08-23

**Authors:** Gail M Seigel, Dhaval K Shah, Pia Mendoza, Ezster Szalai, Hans Grossniklaus, Yinghui Song, Jidong Shan

**Affiliations:** ^1^ University at Buffalo, Center for Hearing and Deafness, Buffalo, NY; ^2^ University at Buffalo, Department of Pharmaceutical Sciences, Buffalo, NY; ^3^ Emory Eye Center, Emory University, Atlanta, GA; ^4^ Molecular Cytogenetic Core, Albert Einstein College of Medicine, NY

**Keywords:** Retinoblastoma, Her2, Adjacent tissues

## Abstract

Retinoblastoma (RB) is an ocular tumor of early childhood. Current treatments attempt to preserve visual function, but may spare chemoresistant tumor cells. One potential therapeutic target for RB is HER2, (ERBB2), expressed in RB in truncated form. In this study, we tested the hypothesis that *Her2* DNA and RNA are expressed in RB tumors and adjacent retina. We examined 24 human RB tumors as well as normal-appearing adjacent retinal tissues for Her2 DNA and RNA expression by *in situ* hybridization. We also examined 28 RB tumors for HER2 protein immunoreactivity. 21/22 RB tumors expressed *Her2* DNA and 14/19 tumors expressed *Her2* RNA. In 17 paired cases, there were three cases in which *Her2* DNA was detected, but not RNA. We also saw *Her2* RNA signal in six instances of “normal” adjacent retinal tissue. Heterogeneous HER2 protein expression in specific tumor regions also was confirmed by quantitative HER2 immunohistochemistry. In summary, *Her2* DNA and RNA are expressed in many RB tumors, and in some adjacent ocular tissues, with hetereogenous protein expression throughout. These results may provide important insights regarding RB tumor progression, and drug targeting approaches designed to spare the eye, preserve vision and improve quality of life for RB patients.

## INTRODUCTION

Human epidermal growth factor receptor 2 (HER2, ERBB) is a cancer biomarker and drug target encoded on human chromosome 17q21–22 [1]. It is expressed in a variety of malignancies [2, 3, 4, 5] and is associated with a poor prognosis [6]. Recently, our group reported on HER2 expression in retinoblastoma, an ocular tumor of childhood [7]. This was an unexpected finding at the time, since a previous study had suggested that HER2 would not be expressed in RB [8]. This negative prediction was based on immunohistochemistry of RB tumors using only one antibody [8]. Our results provided a potential explanation for this inconsistency, as the HER2 expressed in retinoblastoma appeared to be a truncated protein, not detectable by some antibodies directed against the missing portions of the protein [7].

Our 2016 study demonstrated HER2 expression in retinoblastoma tumors (and its absence in normal ocular tissues) by multiple methods, including immunohistochemistry, flow cytometry, RT-PCR and western immunoblot [7], but not in situ hybridization. This study focused on the tumor itself, without analysis of adjacent retinal tissues. A subsequent study by Sousa and colleagues questioned the extent of HER2 expression in retinoblastoma, as the protein was immunonegative in 49/60 retinoblastoma cases and very low in 11/60 cases that they tested [9]. Since the Sousa study involved one of the same antibodies used in our study (Sigma, HPA001383), the simplest explanation for this conflicting result was a difference in methodology (shorter incubation periods, different detection methods).

Our present study demonstrates new and important characteristics of Her2 expression in retinoblastoma by analyzing DNA and RNA expression in retinoblastoma tumors, including adjacent retinal tissue, as well as regional Her2 immunoreactivity within the tumor itself. To this end, we examined 24 retinoblastoma tumors and adjacent ocular tissue by fluorescent and colorimetric *in situ* hybridization, specifically DNA-FISH and RNA-CISH. We followed up with additional HER2 immunohistochemistry of 28 RB tumors in different zones (central tumor, transitional zone, leading edge, vitreous seeds) to assess regional differences in HER2 immunoreactivity. Positive results in RB tumors and adjacent tissues reveal the utility of FISH-DNA and CISH-RNA analysis in assessing *Her2* expression in retinoblastoma for potential drug targeting approaches and studies of tumor progression.

## RESULTS

For FISH and CISH analyses, a total of 24 retinoblastoma cases were examined, with 17 paired cases that were tested for both FISH and CISH. A summary of these results is shown in Table 1, with the unpaired cases shaded. For FISH, a total of 21/22 RB tumors expressed some Her2 DNA, while 14/19 RB tumors expressed some Her2 RNA by CISH. For direct comparison, 17 paired RB cases were tested for both FISH and CISH; in three of these cases *Her2* DNA was detected, but not RNA.

**Table 1:** Her2 FISH and CISH in retinoblastoma tumors and control tissues Her2 FISH and CISH were performed according to Methods, with individual results for each tumor sample, alongside clinical information when available. Shaded rows indicate tumors that were not paired for both FISH and CISH

Sample	Staging	Her2 FISH DNA	Her2 CISH RNA
Array A 1,2 RB	T3cN0M0	low	low
Array A 3,4 RB	T4bN0M0	+	−
Array A 5,6 RB	T2N0M0	+	+
Array A 7,8 RB	T2N0M0	+	+
Array B 1,2 RB	T3cN0M0	+	+
Array B 3,4 RB	T2N0M0	+	+
Array B 5,6 RB	T3aN0M0	+	+
Array B 7,8 RB	T2N1M0	+	+
Array C 1,2 RB	T3N0M0	+	+
Array C 3,4 RB	T2N0M0	+	+
Array C 5,6 RB	T2N0M0	+	−
Array C 7,8 RB	T3bN0M0	+	+
Array D 1,2 RB	T3aN0M0	low	low
Array D 3,4 RB	TIN0M0	+	−
RB2	T3bpNXcM0	+	N/A
RB4	T3bNXMX	N/A	−
RB5	T2bpNXpMX	+	N/A
RB6	T3bNXMX	+	N/A
RB10	T2aN0MX	−	−
RB11	T3bNXM	N/A	+
RB17	T1NXMX	+	N/A
RB18	T3bNXMX	+	N/A
RB19	T1NXMX	+	+
RB20	T3bNXMX	+	+
**Sample**	**Staging**	**Her2 FISH DNA**	**Her2 CISH RNA**
Her+ xenograft (pos control)	N/A	+	+
Adrenal tumor (neg control)	N/A	−	−

### Visualization of Her2 DNA-FISH in RB

**Figure 1:**
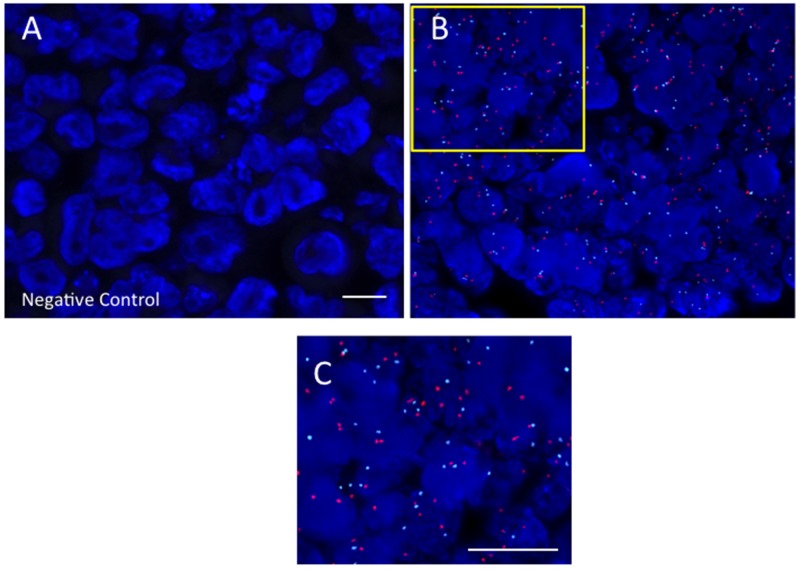
Her2 DNA expressed in RB tumors Fluorescence in situ hybridization (FISH) images of Her2 DNA signal (red) in paraffin embedded retinoblastoma tumor samples. Panel A: Negative control probe; Panel B: Retinoblastoma tumor with red Her2 signal, with the yellow square shown as a higher magnification inset [Panel C]. Scale bars = 5 microns.

DNA-FISH results were visualized by fluorescent microscopy, with representative images shown in Figure 1. *Her2* signals (red) are visible in panels B and C that contrast with the negative control probe image in panel (A). Although there was heterogeneity of *Her2* DNA expression among the tumors examined, there was clear Her2 DNA expression in 22/23 of tumors tested. The only *Her2* negative tumor by DNA-FISH was tumor RB10, staged as T2aN0MX, with minimal tumor spread.

### Visualization of Her2 RNA-CISH in RB

**Figure 2:**
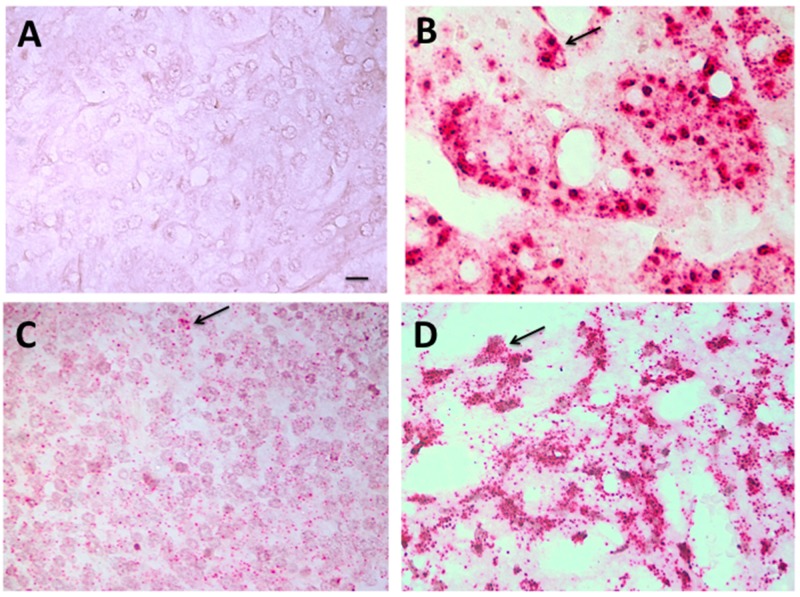
Her2 RNA expressed in RB tumors Colorimetric in situ hybridization (RNA-CISH) images of Her2 RNA signal (red puncta) in paraffin embedded tissue samples. Panel A: Negative control tissue (pheochromocytoma tumor); Panel B: Her+ xenograft; Panel C: Her2 signal in a moderately-expressing retinoblastoma tumor; Panel D: Her2 signal in a high-expressing retinoblastoma tumor. Arrows point to examples of Her2 signal. Scale bar = 5 microns.

RNA-CISH results were visualized by brightfield microscopy, with examples shown in Figure 2. A *Her2* negative adrenal gland tumor (panel A) and a positive control Her2+ xenograft (panel B) are seen in comparison with *Her2*-expressing RB tumors (low-moderate, Panel C; high, Panel D). *Her2* RNA expression was detected in 14/19 RB tumors tested, with some heterogeneity within the tissues. In 17 paired cases of RB tested for both DNA-FISH and RNA-CISH, there were three cases in which we did not detect *Her2* RNA, although these samples did express *Her2* DNA as shown in Table 1.

### Her2 expression in adjacent retina and optic nerve

**Figure 3:**
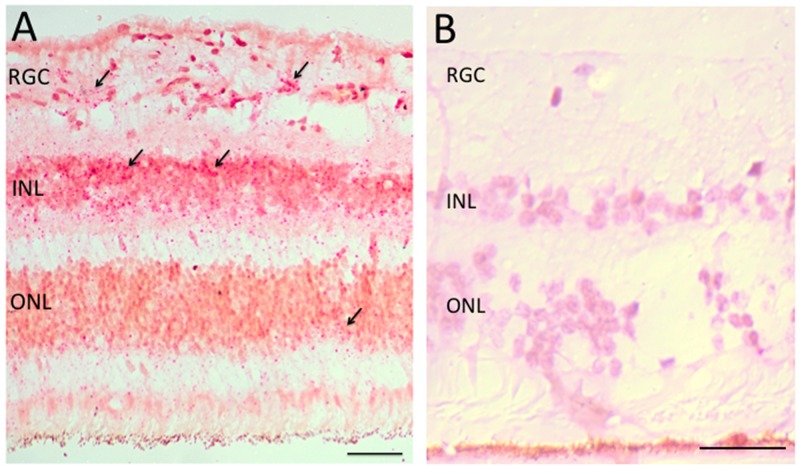
Her2 RNA expression in normal adjacent retina Panel A: Her2 RNA, as detected in retinal tissue adjacent to retinoblastoma tumor by RNAScope colorimetric in situ hybridization (RNA-CISH). Red dots indicate Her2 signals. Arrows show examples of Her2 signal. Panel B: Example of retinal tissue adjacent to retinoblastoma tumor that does not exhibit Her2 signal. ONL= outer nuclear layer; INL = inner nuclear layer; RGC = retinal ganglion cell layer. Scale bar = 20 microns.

**Figure 4:**
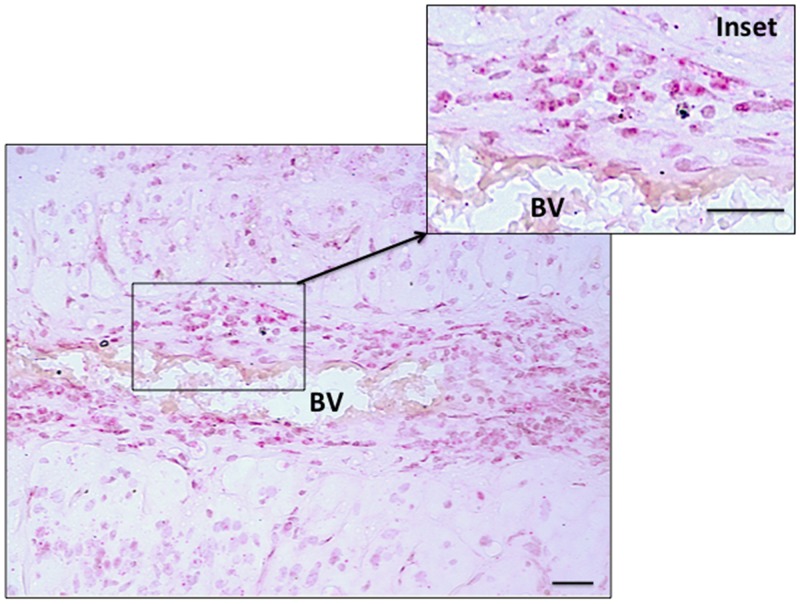
Her2 RNA expression in optic nerve of tumor-bearing eye Her2 RNA adjacent to a blood vessel (BV) in the optic nerve of tumor-bearing eye detected by RNAScope colorimetric in situ hybridization (RNA-CISH). Red dots indicate Her2 signals. Inset shows higher magnification. Scale bars = 20 microns.

Histologically normal tissue adjacent to a tumor is often used as control tissue in comparative tumor studies. However, little is known about the characteristics of this adjacent tissue, how it is influenced by the tumor, and how it compares with non-tumor-bearing tissues. We took this opportunity to examine normal adjacent retinal tissue in these RB cases to determine whether we could detect *Her2*. In six separate RB cases, we observed *Her2* DNA and/or RNA expression in the adjacent retina, one example of which is shown in Figure 3. These *Her2* signals were predominantly localized to the inner nuclear layer (INL) and outer nuclear layer (ONL) of the histologically normal adjacent retina, although we did see some signal in the ganglion cell layer as well. We also saw an example of increased *Her2* signal in optic nerve adjacent to a blood vessel (BV), as shown in Figure 4.

### Immunoreactivity of HER2 in various regions of the tumor

**Figure 5:**
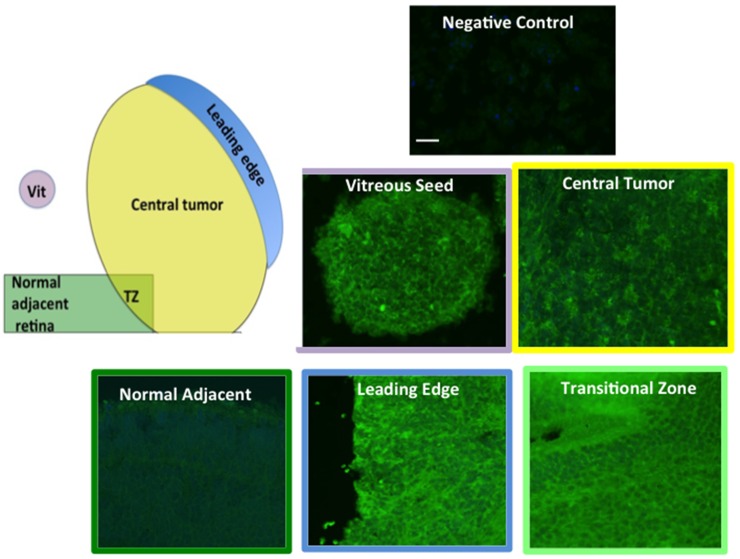
HER2 IHC staining RB tumors were immunostained for HER2 as described in Methods. Diagram indicates regions that were examined. Examples of HER2 immunostaining are shown: Negative control, Central tumor, Vitreous Seed (Vit), Leading Edge, Transitional Zone (TZ), Adjacent Normal. Scale bar = 10 microns

**Figure 6:**
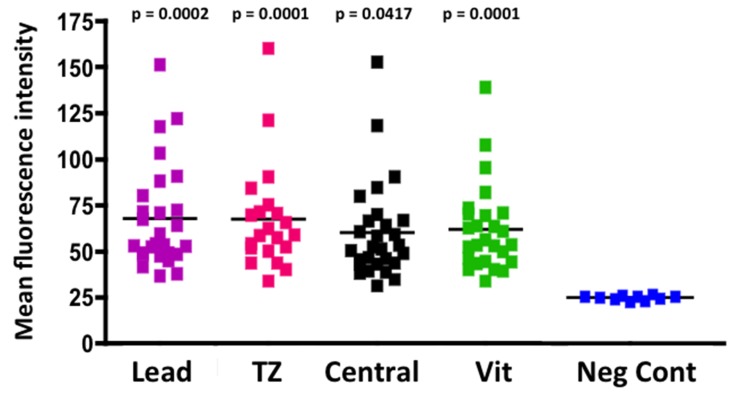
Immunofluorescence measurements of RB tumor regions RB tumors were immunostained for HER2 and fluorescence intensity analyzed by Image J as described in Methods. Fluorescence intensity for each region (Y axis) was compared with negative controls (Lead= leading edge, TZ= transition zone, Central= central tumor, Vit = vitreous seed, Neg Cont= negative control. P values when comparing each region with the negative control ranged from 0.0001 to 0.0417 as shown in the graph. Horizontal solid black line indicates mean fluorescence for each group.

In order to correlate FISH and CISH analysis with protein expression, (particularly in the transitional zone between tumor and adjacent tissue), we examined 28 RB tumors for HER2 immunoreactivity in various regions of the tumor, including the leading edge, central tumor, vitreous seeds and transitional zone between the tumor and adjacent tissue. Examples of these regions are illustrated in Figure 5. We analyzed these images with Image J to assess fluorescence intensity. Normal adjacent retinal tissue was not included in ImageJ analysis, as there were not enough cases or areas of clear-cut normal adjacent tissue to be able to determine statistical significance. In general, however, we did not see intense HER2 immunoreactivity in normal adjacent retina (example shown in Figure 5). Of the 28 RB tumors examined, 14 were brighter than 2 standard deviations above the negative control, with p values ranging from 0.0001 to 0.0417 (Figure 6). All regions assessed were statistically more fluorescent than negative controls, with heterogeneity between individual tumors and within each tumor section. When we compared mean fluorescence intensity between the different HER2-stained tumor regions with one another, there was no significant difference.

## DISCUSSION

### DNA-FISH vs. RNA-CISH and protein expression

We utilized both fluorescent and colorimetric *in situ* hybridization as a means to localize *Her2* DNA and RNA in retinoblastoma tumors and adjacent tissues. In general, RNA *in situ* hybridization for *Her2* has a strong correlation with FISH-DNA analysis and immunohistochemistry [12]. Interestingly, there was not complete concordance between our DNA-FISH and RNA-CISH results. Table 1 indicates that in 17 paired cases of RB tested for both DNA-FISH and RNA-CISH, there were three cases in which *Her2* DNA was expressed but not *Her2* RNA. This is not entirely unexpected, since RNA is a downstream nucleic acid product that is subject to transcriptional regulation [13]. Although RNA is less stable than DNA in fixed tissue [14], the tumor array cores were prepared and cut within a few weeks of analysis. Therefore, transcriptional regulation is the most likely explanation for the three RB cases that expressed *Her2* DNA but not RNA.

In addition to CISH and FISH, we examined 28 RB tumors for HER2 immunoreactivity in various regions of the tumor, including in the transitional zone between tumor and adjacent retinal tissue. Half of the tumors were considered HER2 immunoreactive (at least two standard deviations of brightness above the negative control), a smaller percentage of IHC positive tumors compared with FISH or CISH. This difference is likely due to the fact that i) these IHC tumors were not matched with FISH and CISH tumors due to tissue availablity and ii) protein expression of HER2 relies upon not only DNA expression, but also RNA expression and translation of mRNA into protein. So, it is not surprising that there would be fewer tumors immunoreactive for HER2 than Her2 positive for FISH or CISH. However, the immunoreactivity of 14/28 RB tumors using the Sigma, HPA001383 antibody, in conjunction with our CISH and FISH data, reinforce our hypothesis that *Her2* is expressed in many retinoblastoma tumors at the DNA, RNA and protein levels.

### Her2 expression in normal adjacent tissue

Histologically normal tissues adjacent to a tumor are often designated as “healthy control tissues” for comparative cancer studies, with the assumption that a histologically normal morphology translates to normal cell behavior. However, normal-appearing tissues adjacent to tumors may express abnormal transcriptomes [15], or exhibit other atypical characteristics, such as activated inflammatory and immune responses [16]. In human breast cancer, altered Her2 expression has been detected in 5% of peritumoral tissues [17].

In our study of RB adjacent retinal tissue, *Her2* expression is evident prior to histological transformation, particularly in the inner and outer nuclear layers of the retina. This result is especially intriguing, since live imaging of RB tumors in patients shows that early lesions are visible in the inner nuclear layer of the retina [18] and suggests that the elusive RB “cell of origin” may arise from the middle layers of the retina [19], in the same regions where we saw *Her2* RNA expression in normal-appearing tissues. We also saw increased *Her2* RNA signal in cells surrounding a blood vessel in the optic nerve of a tumor-bearing eye, which may be evidence of ongoing metastasis [20]. Importantly, our earlier study of normal human retina (without retinoblastoma) showed no Her2 expression, either by immunohistochemistry or by qRT-PCR [7]. Therefore, the expression of *Her2* in tumor-adjacent, morphologically normal retinal tissue and optic nerve of tumor-bearing eyes provides a new clue in the study of RB tumor progression and malignant transformation.

### Her2 expression and cancer stem cells

Many tumors contain a small percentage of cancer stem cells, with the ability to self-renew, metastasize, and confer chemoresistance (for review see [21]). Previous work from our group, confirmed by others, has demonstrated the presence of these cancer stem cell markers in human retinoblastoma [22,23,24,25]. We know that in breast cancer, *Her2* expression and overexpression is associated with a cancer stem cell phenotype (for Review, see [26]). *Her2* overexpression is associated with an increased subpopulation of cancer stem cells and correlates with activation of the PI3K/AKT pathway, leading to increased expression of stem cell markers Oct3/4, Notch1, Notch2, Jagged1 and Gli1 [27]. In retinoblastoma, we do not have as much information on the role of *Her2* in the modulation of cancer stem cell behavior. However, we do know that the expression of *Her2*, a truncated protein in retinoblastoma [7], remains intact at the trastuzumab binding site, with potential as a target for anti-HER2 therapy [28]. The expression of *Her2* in adjacent retina and optic nerve may be relevant to the progression of retinoblastoma, and the effect of the tumor microenvironment on surrounding retinal tissues. In turn, our findings on *Her2* expression in RB and adjacent tissues may aid in our development of strategies to target *Her2* as a viable drug target or prognostic indicator in retinoblastoma.

## METHODS

### Tissue Samples

All human tissues in this study came from de-identified pathology cases and were considered exempt as defined by the Common Rule 45 CFR 46.102. For all experiments, paraffin-embedded human retinoblastoma samples were assessed from two sources: a repository at the Emory University Ocular Pathology Lab, and a retinoblastoma tissue array BC35111a (US Biomax, Derwood, MD). Control slides included a Her+ xenograft, and a Her2 negative phaeochromocytoma tumor that was part of the tissue array.

### Her2 FISH

The *Her2* FISH method was used to detect *Her2* DNA in retinoblastoma tissues, “normal” adjacent retina, along with control tissues. Paraffin slides were baked at 60ºC overnight and treated with Citrisolv (Fisher Scientific, Pittsburgh, PA) three times for 10 min at room temperature, followed by 100% ethanol, twice for 10 min at room temperature and allowed to air dry. The slides were incubated in pretreatment buffer (Vysis Paraffin Pretreatment Solution, 30-80600 which is from Vysis Paraffin Pretreatment Reagent Kit, 32-801200, Abbott Des Plaines, IL) at 80º C for 30 min. The slides were rinsed in washing buffer (2x SSC) at room temperature for 5 min, and then transferred to ddH2O at room temperature for 5 min. The slides were then treated with protease (Vysis protease, 30-806001) at 37ºC for 15 min, rinsed in washing buffer (2x SSC) at room temperature for 5 min, followed by ddH2O, at room temperature for 5 min. The slides were taken through a series of alcohols (70%, 90% and 100% EtOH) for 3 min each and allowed to air dry. Co-denaturation of the slide and probe was performed at 76ºC for 5 min. The *Her2* BAC clone probe (RP11-1065L22, RP11-449M14, made in-house) (Roohi, et al., 2008) was hybridized at 37ºC overnight, then rinsed in 4x SSC/0.1% Tween 20. This was followed by a series of washes: 2xSSC/0.3% Nonidet-P40 at 72ºC for 2 min, 4xSSC/0.1% Tween-20 at room temperature for 3 min, and a final series of alcohols (70%, 90% and 100% EtOH), 3 min each. The slides were allowed to air dry and coverslipped with DAPI/antifade mounting medium (Applied Spectral Imaging, Carlsbad, CAFPRPR0006).

### HER2 CISH

For colorimetric in situ hybridization of RNA (RNA-CISH), we used a highly specific RNAScope Fast Red kit (Advanced Cell Diagnostics, Newark, CA) and Her2/Erbb2 human probe (#310081, RNAscope Probe - Hs-ERBB2; Advanced Cell Diagnostics) to detect RNA. For a positive control human probe, we used ubiquitin (#310041, RNAscope® Positive Control Probe - Hs-UBC, Advanced Cell Diagnostics). The RNA *in situ* method was carried out according to the RNAScope kit instructions, with a two hour probe hybridization at 40ºC.

### HER2 immunoreactivity

For immunohistochemistry, we examined 28 paraffin-embedded RB tumors using a rabbit anti-HER2 antibody (Sigma, HPA001383) according to our standard protocol [11]. Due to availability of tissue sections, these immunohistochemistry samples were not paired with the CISH and FISH samples. Briefly, we baked paraffin slides for 60 minutes at 60°C, incubated the sections with a series of xylene and alcohols, boiled in antigen retrieval solution (sodium citrate, pH 6.0), and blocked for fifteen minutes at room temperature in 1% bovine serum albumin, 0.5% Triton-X100 in PBS. Slides were incubated in anti-Her2 primary antibody (Sigma HPA001383) at 5ug/ml (or isotype control antibody) for one hour at room temperature. Slides were washed three times in PBS and then incubated in FITC conjugated anti-rabbit secondary antibody (Sigma, stock concentration listed as ranging from 3–6.5 mg/mL), diluted 1:400 in PBS. Slides were cover-slipped and photographed with a Nikon E600 microscope (Melville, NY) equipped with a Spot digital camera and Spot Advanced software (Sterling Heights, MI). We measured fluorescence staining intensity using Image J software, with three image samplings per section in the regions being measured. Some tumor sections did not have discernable regions for every parameter being measured, which accounts for the varying number of spots on each graph. Fluorescence intensities were averaged for each measurable region of the tumor, compared with fluorescence intensities of negative control images in that region, analyzed by one way ANOVA and graphed using Prism (Graphpad, San Diego, CA) software.
